# Supplementary material for: Aerobic Degradation Characteristics of Decabromodiphenyl ether through Rhodococcus ruber TAW-CT127 and Its Preliminary Genome Analysis
Source: Microorganisms. 2022 Jul 17;10(7):1441. doi: 10.3390/microorganisms10071441 (PMC9319644; doi:10.3390/microorganisms10071441)
Supplement: Supplementary file 1 [file microorganisms-10-01441-s001.zip › microorganisms-1771399-supplementary/Table S1.pdf]

Table S1. 16S rRNA sequence of strain TAW-CT127

16S rRNA sequence of strain TAW-CT127
TCCTGGCTCAGGACGAACGCTGGCGGCGTGCTTAACACATGCAAGTCGAACGATG<br>AAGCCCAGCTTGCTGGGTGGATTAGTGGCGAACGGGTGAGTAACACGTGGGTGAT<br>CTGCCCTGCACTTCGGGATAAGCCTGGGAAACTGGGTCTAATACCGGATAGGACCT<br>CGGGATGCATGTTCCGGGGTGGAAAGGTTTTCCGGTGCAGGATGGGCCCGCGGCCT<br>ATCAGCTTGTTGGTGGGGTAACGGCCCACCAAGGCGACGACGGGTAGCCGGCCTG<br>AGAGGGCGACCGGCCACACTGGGACTGAGACACGGCCCAGACTCCTACGGGAGG<br>CAGCAGTGGGGAATATTGCACAATGGGCGCAAGCCTGATGCAGCGACGCCGCGTG<br>AGGGATGACGGCCTTCGGGTTGTAAACCTCTTTCAGTACCGACGAAGCGCAAGTG<br>ACGGTAGGTACAGAAGAAGCACCGGCCAACTACGTGCCAGCAGCCGCGGTAATA<br>CGTAGGGTGCGAGCGTTGTCCGGAATTACTGGGCGTAAAGAGCTCGTAGGCGGTTT<br>GTCGCGTCGTCTGTGAAAACCCGCAGCTCAACTGCGGGCTTGCAGGCGATACGGG<br>CAGACTTGAGTACTGCAGGGGAGACTGGAATTCCTGGTGTAGCGGTGAAATGCGC<br>AGATATCAGGAGGAACACCGGTGGCGAAGGCGGGTCTCTGGGCAGTAACTGACGC<br>TGAGGAGCGAAAGCGTGGGTAGCGAACAGGATTAGATACCCTGGTAGTCCACGCC<br>GTAAACGGTGGGCGCTAGGTGTGGGTTTCCTTCCACGGGATCCGTGCCGTAGCTAA<br>CGCATTAAGCGCCCCGCCTGGGGAGTACGGCCGCAAGGCTAAAACTCAAAGGAAT<br>TGACGGGGGCCCGCACAAGCGGCGGAGCATGTGGATTAATTCGATGCAACGCGAA<br>GAACCTTACCTGGGTTTGACATACACCGGACCGCCCCAGAGATGGGGTTTCCCTTG<br>TGGTCGGTGTACAGGTGGTGCATGGCTGTCGTCAGCTCGTGTCGTGAGATGTTGGGT<br>TAAGTCCCGCAACGAGCGCAACCCTTGTCCTGTGTTGCCAGCACGTAATGGTGGGG<br>ACTCGCAGGAGACTGCCGGGGTCAACTCGGAGGAAGGTGGGGACGACGTCAAGTC<br>ATCATGCCCCTTATGTCCAGGGCTTCACACATGCTACAATGGCCGGTACAGAGGGC<br>TGCGATACCGCGAGGTGGAGCGAATCCCTTAAAGCCGGTCTCAGTTCGGATCGGG<br>GTCTGCAACTCGACCCCGTGAAGTCGGAGTCGCTAGTAATCGCAGATCAGCAACG<br>CTGCGGTGAATACGTTCCCGGGCCTTGTACACACCGCCCGTCACGTCATGAAAGTC<br>GGTAACACCCGAAGCCGGTGGCCTAACCCCTCGTGGGAGGGAGCCGTCGAAGGTG<br>GGATCGGCGATTGGGACGAAGTCGTAACAAGGTAGC
