# Supplementary material for: Aerobic Degradation Characteristics of Decabromodiphenyl ether through Rhodococcus ruber TAW-CT127 and Its Preliminary Genome Analysis
Source: Microorganisms. 2022 Jul 17;10(7):1441. doi: 10.3390/microorganisms10071441 (PMC9319644; doi:10.3390/microorganisms10071441)
Supplement: Supplementary file 1 [file microorganisms-10-01441-s001.zip › microorganisms-1771399-supplementary/Table S2.pdf]

Table S2. COG analysis of strain TAW-CT127

Categories	Function annotation	TAW-CT127	
		orf number	percentage(%)
A	RNA processing and modification	1	0.04
B	Chromatin structure and dynamics	1	0.04
C	Energy production and conversion	291	10.22
D	Cell cycle control, cell division, chromosome partitioning	21	0.74
E	Amino acid transport and metabolism	250	8.78
F	Nucleotide transport and metabolism	66	2.32
G	Carbohydrate transport and metabolism	166	5.83
H	Coenzyme transport and metabolism	94	3.30
I	Lipid transport and metabolism	334	11.74
J	Translation, ribosomal structure and biogenesis	146	5.13
K	Transcription	218	7.66
L	Replication, recombination and repair	120	4.22
M	Cell wall/membrane/envelope biogenesis	102	3.58
N	Cell motility	0	0.00
O	Posttranslational modification, protein turnover, chaperones	91	3.20
P	Inorganic ion transport and metabolism	176	6.18
Q	Secondary metabolites biosynthesis, transport and catabolism	184	6.47
R	General function predictiononly	235	8.26
S	Function unknown	198	6.96
T	Signal transduction mechanisms	92	3.23
U	Intracellular trafficking, secretion, and vesicular transport	18	0.63
V	Defense mechanisms	42	1.48
W	Extracellular structures	0	0.00
Y	Nuclear structure	0	0.00
Z	Cytoskeleton	0	0.00
**Total**		**2846**	**100**
